# IL-2 availability regulates the tissue specific phenotype of murine intra-hepatic Tregs

**DOI:** 10.3389/fimmu.2022.1040031

**Published:** 2022-10-31

**Authors:** Ada S. Kurt, Karoline Strobl, Paula Ruiz, Gabriel Osborn, Tonika Chester, Lauren Dawson, Karsten M. Warwas, Elizabeth H. Grey, Sotiris Mastoridis, Elisavet Kodela, Niloufar Safinia, Alberto Sanchez-Fueyo, Marc Martinez-Llordella

**Affiliations:** ^1^ Institute of Liver Studies, Division of Transplantation Immunology & Mucosal Biology, King’s College London, London, United Kingdom; ^2^ Institute of Cancer Research, Medical University of Vienna, Vienna, Austria; ^3^ Applied Tumour Immunity, German Cancer Research Centre (DKFZ), Ruprecht-Karls-Universitat, Heidelberg, Germany; ^4^ Nuffield Department of Surgical Sciences, University of Oxford, Oxford, United Kingdom

**Keywords:** regulatory T cells (Tregs), liver, acute inflammation, Treg depletion, CCl₄ induced liver injury, tissue specific

## Abstract

CD4+CD25+Foxp3+ Tregs are known to acquire tissue-specific features and exert cytoprotective and regenerative functions. The extent to which this applies to liver-resident Tregs is unknown. In this study, we aimed to explore the phenotypic and functional characteristics of adult murine liver resident Tregs during homeostasis. Additionally, we investigated their role in ameliorating liver inflammation and tissue damage. Quantification of Foxp3+CD4+CD25+ cells comparing different tissues showed that the liver contained significantly fewer resident Tregs. A combination of flow cytometry phenotyping and microarray analysis of intra-hepatic and splenic Tregs under homeostatic conditions revealed that, although intra-hepatic Tregs exhibited the core transcriptional Treg signature, they expressed a distinct transcriptional profile. This was characterized by reduced CD25 expression and increased levels of pro-inflammatory Th1 transcripts *Il1b* and *Ifng*. *In vivo* ablation of Tregs in the Foxp3-DTR mouse model showed that Tregs had a role in reducing the magnitude of systemic and intra-hepatic inflammatory responses following acute carbon tetrachloride (CCl₄) injury, but their absence did not impact the development of hepatocyte necrosis. Conversely, the specific expansion of Tregs by administration of IL-2 complexes increased the number of intra-hepatic Tregs and significantly ameliorated tissue damage following CCl₄ administration in C57BL/6 mice. The cytoprotective effect observed in response to IL-2c was associated with the increased expression of markers known to regulate Treg suppressive function. Our results offer insight into the transcriptome and complex immune network of intra-hepatic Tregs and suggest that strategies capable of selectively increasing the pool of intra-hepatic Tregs could constitute effective therapies in inflammatory liver diseases.

## Introduction

Forkhead box P3 positive (Foxp3+) regulatory T cells (Tregs) are a subset of T helper cells with immunosuppressive properties that are central to the maintenance of immune homeostasis and peripheral tolerance, both in mice and humans, and are being developed as novel immunomodulatory therapies for autoimmune diseases and transplantation (1, 2). Due to their constitutive expression of CD25, the IL-2 receptor alpha chain (IL-2RA), Tregs respond to even very low concentrations of IL-2, a cytokine secreted by effector T cells (Teffs) (3) that is essential for Treg survival and suppressive function *via* STAT5 signalling (4). Furthermore, the administration of exogenous IL-2, either in the form of low dose recombinant IL-2 (LDIL-2) therapy in humans or as IL-2 complexes (IL-2c) consisting of soluble IL-2 conjugated with the IL-2 specific monoclonal antibody JES6-1A12, has been shown to increase the pool of Tregs *in vivo* and exert anti-inflammatory effects (5–7).

While all Tregs maintain common gene signatures attaining peripheral tolerance, they are far from being a homogenous cell lineage (8, 9). Thus, increasing evidences indicate that Tregs acquire unique phenotypic features depending on the tissue compartment they reside in (10). Ablation of Foxp3-expressing cells in animal models of lung, skeletal muscle, heart muscle, skin, bone, and central nervous system injury shows that the tissue-resident Tregs exhibit distinct cytoprotective and pro-regenerative properties (11–17). Differential tissue-specific features of Tregs include active involvement in cell functional reprogramming, such as the inhibition of M1 macrophage inflammatory activity and the promotion of M2 macrophages polarisation (18). This is because tissue-resident Tregs express the epidermal growth factor family member amphiregulin (Areg) which is known to promote tissue repair and regeneration under inflammatory conditions. Distinct from signals eliciting suppressor function, Areg production is independent of T-cell receptor (TCR) engagement and induced by IL-33 or IL-18 released from activated endothelial cells in response to tissue injury and inflammation. As tissue resident Tregs respond directly to alarmins like IL-33, they play a reparative role in ST2/IL-33/Areg mediated regeneration and differentiation following tissue injury (19, 20). Different non-lymphoid tissue resident Treg populations that display adaptation to tissue microenvironments were reported. For instance, visceral adipose tissue Tregs exhibit distinct phenotype exhibiting high levels of chemokine receptors CCR1, CCR2, CCR6 and CCL6 and play a regulatory role in insulin resistance and sensitivity (21, 22). On the other hand, muscle Tregs exhibit higher expression of CTLA-4, TIM-3 and ST2, as well as chemokine receptor CCR1 and activate satellite cell proliferation and muscle regeneration through Areg (11, 12).

The liver is an organ with unique regenerative capacity, which is key to ensure that hepatic functions are maintained following acute tissue damage/loss (23). A previous study characterised the transcriptomic profile of thymus-derived Tregs in neonatal livers from 1–2-week-old-mice under homeostatic conditions and showed that their accumulation is critical to maintain self-tolerance and liver maturation (24). However, the extent to which adult mice livers harbour tissue-resident Tregs with distinct phenotypic characteristics has not been previously investigated. Likewise, the broader functional role of intra-hepatic Tregs in the initiation and modulation of liver inflammation remains to be fully explored. To answer these questions, in the current study we aimed to delineate the specific adaptations of liver resident Tregs by analysing their molecular profile during homeostatic conditions. Further, using the well-described acute liver inflammation model induced by the hepatotoxin carbon tetrachloride (CCl₄), we investigated the local and systemic effects of both Treg depletion and Treg augmentation *via* IL-2c administration. Our results highlight how the abundancy and distinct phenotype of intra-hepatic Tregs influence their ability to modulate liver inflammation and they suggest that therapies selectively increasing the intra-hepatic Treg pool could be exploited to regulate inflammatory liver diseases.

## Materials and methods

### Animals

All procedures and experiments were approved by the Animal Welfare and Ethical Review Body of King’s College London. All animals had unrestricted access to food and water and were kept according to the standards of the Animals (Scientific Procedures) Act 1986. Wild-type C57BL/6 mice were purchased from Harlan and kept in our specific pathogen-free animal facility at King’s College London. Foxp3-tm4(YFP/icre) Ayr (Foxp3/YFP-cre) and Foxp3-tm3(DTR/GFP)Ayr (Foxp3-DTR) mice were provided as a kind gift of G. Lombardi, King’s College London. All transgenic mice were bred in-house for the experiments. 8-weeks old male mice were used for all experiments to avoid gender differences.

### Statistics

Unless otherwise stated, statistical analyses were performed with GraphPad Prism 8.0 software. Student’s t test was used for comparison between two groups, and one or two-way ANOVA analysis with Tukey’s *post hoc* correction for pairwise comparisons was used to compare more than two groups (*P < 0.05, **P < 0.01, ***P < 0.001, and ****P < 0.0001). Results are reported as mean ± SEM.

### CCL₄ acute inflammation model

Induction of acute inflammatory liver damage by the hepatotoxin CCL₄ is a widely reported model due to its hepatotoxic capacities impairing key cellular processes and leading to fatty degeneration and steatosis as an effect of trichloromethyl radical metabolised by the cytochrome p450.

### Treg depletion model

DTR-eGFP mice express a diphtheria toxin receptor fused to an enhanced green fluorescent protein) transgene under the control of the FOXP3 promoter to enable the specific depletion of Tregs at any desired time point by application of diphtheria toxin (DT).

### IL-2c, CCl₄, DT treatments

IL-2 complexes (IL-2c) were formed by incubating 1 μg recombinant mouse IL-2 (eBioscience) and 5 μg of purified anti-mouse IL-2 (clone JES6-1A12) (eBioscience) for 30 min at 37°C and were diluted with 75 μl of PBS before injection. Animals received 3 doses of IL-2c overall on consecutive days administered as intraperitoneal injections. Diphtheria toxin (DT) was administered intraperitoneally at a dose of 1μg diluted in 100μl of phosphate-buffered saline on 3 consecutive days. Carbon tetrachloride (CCl₄) was administered intraperitoneally as a single dose (25% CCL₄ in corn oil and 2 μl/g weight of mouse) either 2 days after the last dose of IL-2c or 3 days after the last dose of DT. Mice were sacrificed 24 h after CCL₄ administration by CO_2_ inhalation.

### Isolation of spleen and non-parenchymal intra-hepatic mononuclear cells

Murine spleen and livers were harvested into 1xPBS with 2% FBS. Livers were excised, washed and flushed multiple times with 1xPBS and mechanically homogenised to obtain single cell suspensions (we processed the whole livers in their entirety except for two small fragments employed for RNA extraction and histological analyses, respectively). Previous experiments conducted in our laboratory demonstrated that mechanical homogenisation provided a similar yield of intra-hepatic leukocytes than an alternative method that included liver perfusion and digestion with collagenase (data not shown). Mechanical homogenisation was achieved using a 1 ml syringe plunger and gently passing the tissue through a sterile 70μm cell strainer. Hepatic single-cell suspensions were centrifuged at 100 x g for 2 minutes to eliminate hepatocytes. Cells of the supernatant were retained and washed at 400 x g for 7 minutes and mononuclear cells were isolated by Ficoll^®^-Paque (GE Healthcare) density gradient sedimentation. Spleens were homogenised in a similar manner followed by red blood cell lysis with ammonium chloride potassium (ACK) lysing buffer (Life Technologies) and a wash at 400 x g for 7 minutes.

### RT-PCR analysis

RNA isolation for RT-PCR was prepared by homogenization liver biopsies in TRIzol^®^ Reagent (Ambion by Life Technologies, UK) using Tissuelyser II (Qiagen, UK). The RNA was purified by chloroform and isopropanol extraction and reversed transcribed into cDNA using a High-Capacity cDNA Reverse Transcription kit (Applied Biosystems™, UK) and a T9800 Fast Thermal Cycler (Applied Biosystems, UK). The reaction mixture for SYBR Green RT-PCR assay contained cDNA, 2x Fast SYBR Green PCR master mix (Applied Biosystems, UK) and 10μM of forward and reverse primers (IDT, UK). All amplifications and detections were carried out in a MicroAmp optical 384-well reaction plate covered with an optical adhesive film (Applied Biosystems™, UK) and detected on QuantStudio™ 7 Sequence Detection System (Applied Biosystems™, UK). For statistical analysis, relative fold changes (R) were calculated with the function (R = 2-ΔΔCT), where ΔΔCt is the normalized difference in threshold cycle (Ct) number between the control and test samples. Each CT was calculated from duplicate replicates.

Primer sequences were as follows:

**GAPDH** Forward 5’- CCCATCACCATCTTCCAGGAGC -3’
Reverse 5’- CCAGTGAGCTTCCCGTTCAGC -3’
**TNFa** Forward 5’-CGAGTGACAAGCCTGTAGCC -3’
Reverse 5’-AGATAGCAAATCGGCTGACG -3’
**IL-6** Forward 5’-GAGGATACCACTCCCAACAGACC -3’
Reverse 5’-AAGTGCATCATCGTTGTTCATACA -3’

### Microarray analysis

A small liver biopsy, always from the same lobe to avoid sampling variability, was taken for RNA extraction. RNA extracted from liver biopsies and sorted cells using TRIzol^®^ (Thermo Fisher) was quantified using Qubit HS and its integrity assessed in a Bioanalyzer (Agilent). Samples were then reverse transcribed and amplified before hybridization to Affymetrix Mouse Gene 2.1 ST arrays. Microarray expression data were processed using quantile normalization using the Affy Bioconductor package. A conservative probe-filtering step was conducted next to exclude probes with a coefficient of variation >5%. To identify genes differentially expressed between groups, we used significant analysis of microarray (SAM) statistical significance of differentially expressed transcripts was defined at a fold change higher than 2 and a p value <0.05. To assess the deregulation of sets of genes associated with specific functional pathways, we computed an enrichment score for each of the predefined gene sets included in the MSigDB (https://www.broadinstitute.org/gsea/msigdb/index.jsp) with a p-value <0.05 using Gene set enrichment analysis (GSEA) method using the Gene Ontology (GO) gene sets. All microarray data discussed in this article have been deposited in National Center for Biotechnology Information Gene Expression Omnibus (GEO) (accession no. GSE80814).

### Definition of core Treg transcriptional signature

Pfoertner et al. constructed and validated a unique microarray (Human Treg Chip) containing 350 Treg associated genes based on whole human and mouse genome transcription data in the literature and identified 62 differentially expressed genes in mouse and human Treg cells. We defined the core Treg transcriptional signature using these reported comprehensive set of genes.

### Cell sorting

For cell sorting, Foxp3 ^YFP/cre^ reporter mice were used. Splenocytes and liver single cell suspensions were enriched for CD4+ cells (Stemcell Technologies Cat.# 19772). Following enrichment, samples were counted, washed with PBS and a viability staining was performed using LIVE/DEAD Fixable violet dead cell stain according to manufacturer’s protocol (Life Technologies). Following viability staining, samples were washed with PBS 2% FBS, and the pellets were resuspended in 2% FBS with CD3, CD4 and CD25 antibodies, incubated in the dark, at 4°C for 20 minutes. Samples were then washed, and pellets resuspended in 2% FBS and kept on ice to be sorted and analysed by flow cytometry (FACSAria, BD Biosciences). CD4+CD25+Foxp3^YFP+^ Treg and CD4+CD25-Foxp3^YFP-^ Teff populations were then washed, and cell pellets were transferred to an ultra-high recovery RNAse-free 1.5 ml Eppendorf. After the final wash, the pellets were resuspended in TRIzol (Invitrogen) and were stored in -80°C for subsequent RNA extraction.

### Flow cytometry analysis

Flow cytometry analysis was performed on 1-2 x 10^6^ freshly isolated mouse cells to immunophenotype isolated intra-hepatic and splenic regulatory and effector T cells, macrophages and monocytes. For experiments involving Foxp3-DTR mice, Cells were stained with LIVE/DEAD™ Fixable Violet Dead Cell stain kit (1:1000 dilution with PBS) (Life Technologies). Otherwise, LIVE/DEAD Fixable Green Dead cell stain was used (Life Technologies). Following Live/Dead staining, the cells were washed and resuspended in 100 µl surface staining mastermix (**Table S1**). Intracellular staining was performed using the Foxp3 Fixation/Permeabilization Kit (eBioscience) according to the manufacturer’s instructions. Cells were immunophenotyped using BD LSR Fortessa (BD Bioscience) and analysis was performed using FlowJo software (TreeStar, Inc).

### Cytokine quantification

Immediately prior to sacrifice, mouse blood samples were collected *via* cardiac puncture. Serum was separated from the coagulated blood by centrifugation at 3000 x g for 15 minutes at 4°C. Analysis was performed by mouse ELISA kits for IFN-g, IL-6 and IL-10 (Biolegend, ELISA MAX™). Cytokine concentrations were calculated using linear regression after the generation of a standard curve.

### Serum alanine and aspartate aminotransferase levels

Serum AST and ALT levels were measured using a clinical bioanalyzer (King’s College Hospital). Enzyme activities were shown in international units per litre (IU/L).

### Liver histology

Liver tissue was collected from the same lobe in all mice to avoid sampling variability and placed in 10x formalin and washed in PBS 24 hours later. Fixed liver tissue samples were then placed in plastic cassettes and embedded in paraffin blocks. Liver biopsies were stained with haematoxylin and eosin (H&E). The microscope slides were then analysed under a Nikon Eclipse TE2000 light microscope at 20x magnification.

## Results

### Tissue-resident Tregs are less abundant in the liver than in other organs and exhibit a distinct molecular profile

**Figure 1:**
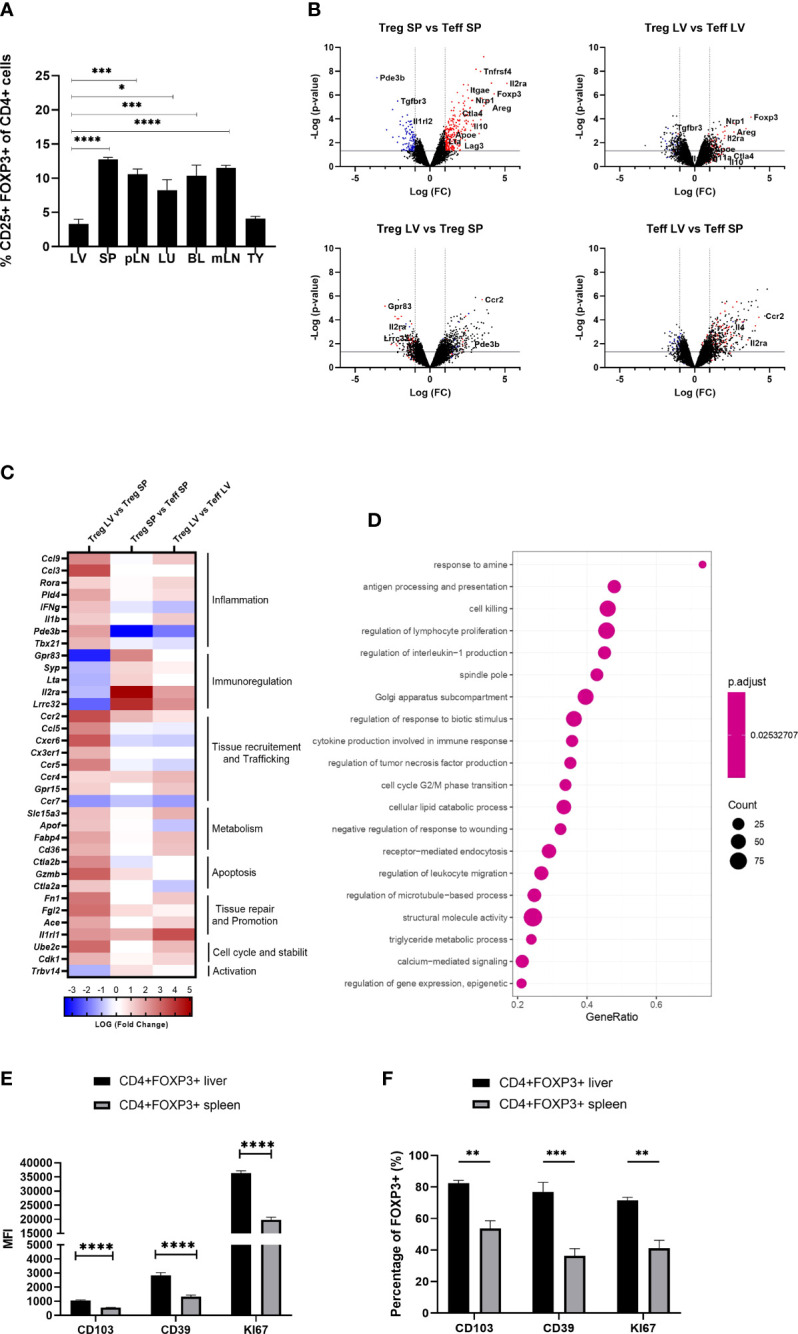
Intra-hepatic Tregs are less abundant but remain to exhibit a core Treg transcriptional signature and have a distinct molecular profile. **(A)** Quantification of the proportion of Foxp3+ CD4+CD25+ cells in the homogenised tissues of liver (LV) (n>8), spleen (SP) (n>8), peripheral lymph nodes (pLN), lung (LU), adipose tissue (AT), blood (BL), mesenteric lymph nodes (mLN) and thymus (TY) in homeostasis (n=4). **(B–D)** Whole genome microarray analysis in intra-hepatic and splenic Tregs versus T-effectors in Foxp3-YFP B6 mice (n=3). **(B)** Volcano plots showing difference in the transcriptome profile of intra-hepatic and splenic Tregs using normalised p-value versus fold change of all 20515 genes and splenic “Treg signature” is defined by up-regulated (red) and down-regulated (blue) genes above p-value of 0.05 and Fold change 2. **(C)** Heatmap exhibits pre-selected differentially expressed genes relevant to Treg function, tissue residency, tissue repair and metabolism between intra-hepatic and splenic Treg population and the significance is determined above p-value of 0.05 and Fold-change 2 **(D)** Gene set enrichment analysis of intra-hepatic Tregs compared with the splenic Tregs. Dot plot shows the top 20 up-regulated GO pathways of biological processes, molecular functions and cellular components with an FDR adjusted q-value of 0.020506. Dot size represents the number of genes enriched in the pathway, and gene ratio represents the ratio of the count of core enrichment genes to count of pathway genes. **(E)** CD4+Foxp3+ cells were gated from homogenised liver and spleen tissue to quantify the MFI of CD103, CD39 and KI67 (n=5). **(F)** Percentage of Foxp3+ cells expressing CD103, CD39 and KI67. Significance was determined by ordinary one-way ANOVA with Sidak’s multiple comparison test for **(A)** and two-way ANOVA with Tukey multiple comparison test for **(E, F)** and the values are shown as the mean ± SEM. For B-D, p<0.05 and Fold Change >2. MFI, geometric mean fluorescence intensity. *P < 0.05, **P < 0.01, ***P < 0.001 and ****P < 0.0001.

Flow cytometric analysis of mononuclear cells isolated from liver, secondary lymphoid organs, blood, thymus, lung and adipose tissue revealed that the liver exhibited the lowest proportion of Foxp3++CD4+CD25+ T cells (3.299 +/- 0.693) (**Figure 1A**). To understand the molecular pathways underpinning this finding, we FACS sorted intra-hepatic and splenic Foxp3+YFP+Tregs of Foxp3-YFP reporter mice and compared their transcriptional profiles using whole-genome transcriptional analysis. While both intra-hepatic and splenic Tregs exhibited a core Treg transcriptional signature, liver Tregs differed from splenic Tregs in the downregulation of immunoregulatory transcripts (*Gpr83, Il2ra, Lrrc32*) while showing upregulation of pro-inflammatory Th1 (IFNg, IL1b) related transcripts (**Figure 1B**). Liver Tregs also exhibited up-regulation of *Pde3b* (cyclic nucleotide phosphodiesterase 3B, CGMP-inhibited), an enzyme required by non-regulatory T cells for survival and expansion (9, 25), and whose transcriptional repression promotes lineage stability and Foxp3 expression in Tregs. The higher expression of *Pde3b* in liver-resident Tregs suggests therefore increased lineage instability as compared to peripheral Tregs. Furthermore, Tregs displayed increased expression of chemokine receptors (*Cxcr6, Cxcr3, Cx3cr1*, *Ccr2, Ccr4*), other tissue homing mediators (*Gpr15*), cell cycle regulators (*Cdk1, Ube2c*), as well as genes associated with effector T cell phenotype (*Il1b, Ifng, Il4, Rora*, *Ccl3)* (**Figure 1C**). Intra-hepatic Tregs also expressed increased levels of transcripts linked to tissue repair and regeneration, such as *Ace* and *Fn1* (both reported to promote regeneration and reparative cell response following ischaemic tissue damage (26, 27)), and *Il1rl1* (IL-33 receptor or ST2). Gene set enrichment analysis (GSEA) confirmed that intra-hepatic Tregs engaged in the regulation of IL-1 and TNF production in Th1 immune responses. Additionally, GSEA showed that the intra-hepatic Treg transcriptome was enriched in pathways involved in the regulation of lymphocyte proliferation, and in metabolic processes such as lipid homeostasis, reactive oxygen species generation and DNA repair (**Figure 1D**).

In agreement with the transcriptional analyses, flow cytometry experiments conducted in parallel showed that, as compared to spleen Tregs, intra-hepatic Tregs exhibited increased expression of tissue-residency marker ITGAE (CD103), high expression of CD39, and high KI67 levels indicating increased proliferation (**Figures 1E**, **F**). Taken together, these data indicate that although intra-hepatic Tregs retain the Treg-specific core transcriptional signature, they exhibit distinct transcriptional and phenotypic features that imply adaptation to the liver tissue microenvironment.

### Depletion of Tregs exacerbates acute liver inflammation but has no effect on the degree of hepatocyte necrosis

**Figure 2:**
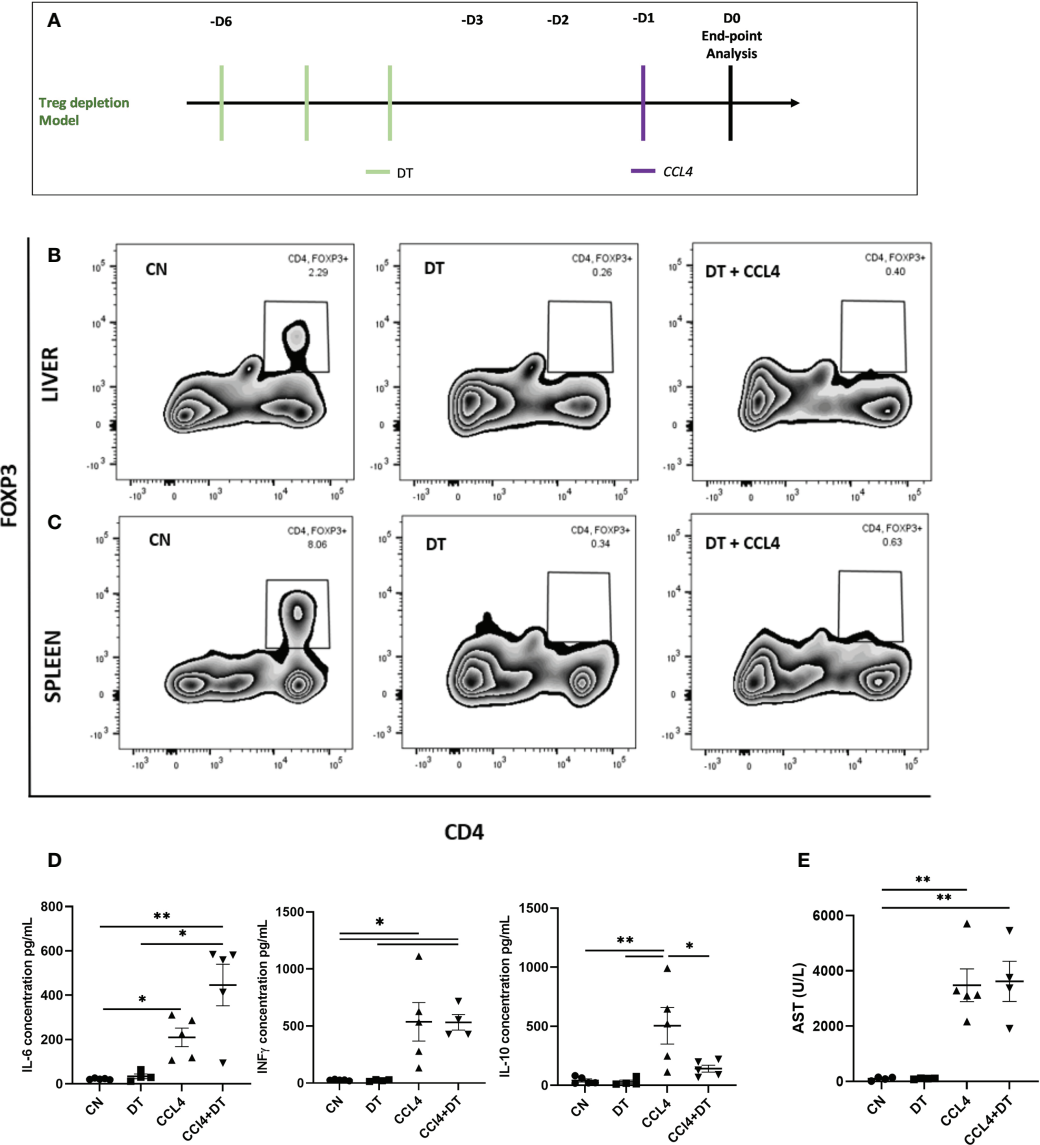
Hepatocyte necrosis is unaltered by Treg depletion. **(A)** Schematic representation of the experimental design for DT induced Treg depletion, 1ug diphtheria toxin (DT) was injected in Foxp3-DTR mice, 3 days prior to CCL₄ administration. Depletion of Foxp3+ cells was analysed by flow cytometry in **(B)** liver and **(C)** spleen and the results are shown as percentages from fixable live/dead stained viable CD4+ cells and representative of 3 individual experiments. **(D)** Serum concentration of IL-6, IFNg and IL-10 cytokines (pg/ml) were measured at 24h in the serum obtained by cardiac puncture following DT and CCL₄ treatment, determined by ELISA (n=5) along with **(E)** AST levels (n=4). The values are shown as the mean ± SEM and one-way ANOVA with Tukey’s multiple comparison test has been performed to show statistical significance, *P < 0.05, **P < 0.01.

Having established that intra-hepatic Tregs exhibit a distinct molecular profile, we next investigated their role in controlling acute inflammatory liver damage induced by the hepatotoxin CCL₄ (**Figure 2A**). We employed DTR-Foxp3 mice, in which DT administration resulted in the almost complete depletion of Tregs both in the liver and the spleen (reduction of Treg percentages from 2.29% to 0.26% in liver and 8.06% to 0.34% in spleen) (**Figures 2B, C**). Following CCL₄ administration, Treg-deficient mice exhibited increased serum levels of IL-6 and reduced levels of IL-10 (**Figure 2D**). The increase in systemic inflammatory mediators was not associated however with an increase in serum aspartate transaminase (AST) as a marker of hepatocyte necrosis (**Figure 2E**). Based on these results, we speculate that Tregs are involved in controlling the systemic effects induced by CCL₄ administration but are not capable of counteracting the direct hepatotoxic effects responsible for hepatocyte necrosis.

### IL-2c expand intra-hepatic Tregs and enhance their expression of immunoregulatory transcripts

**Figure 3:**
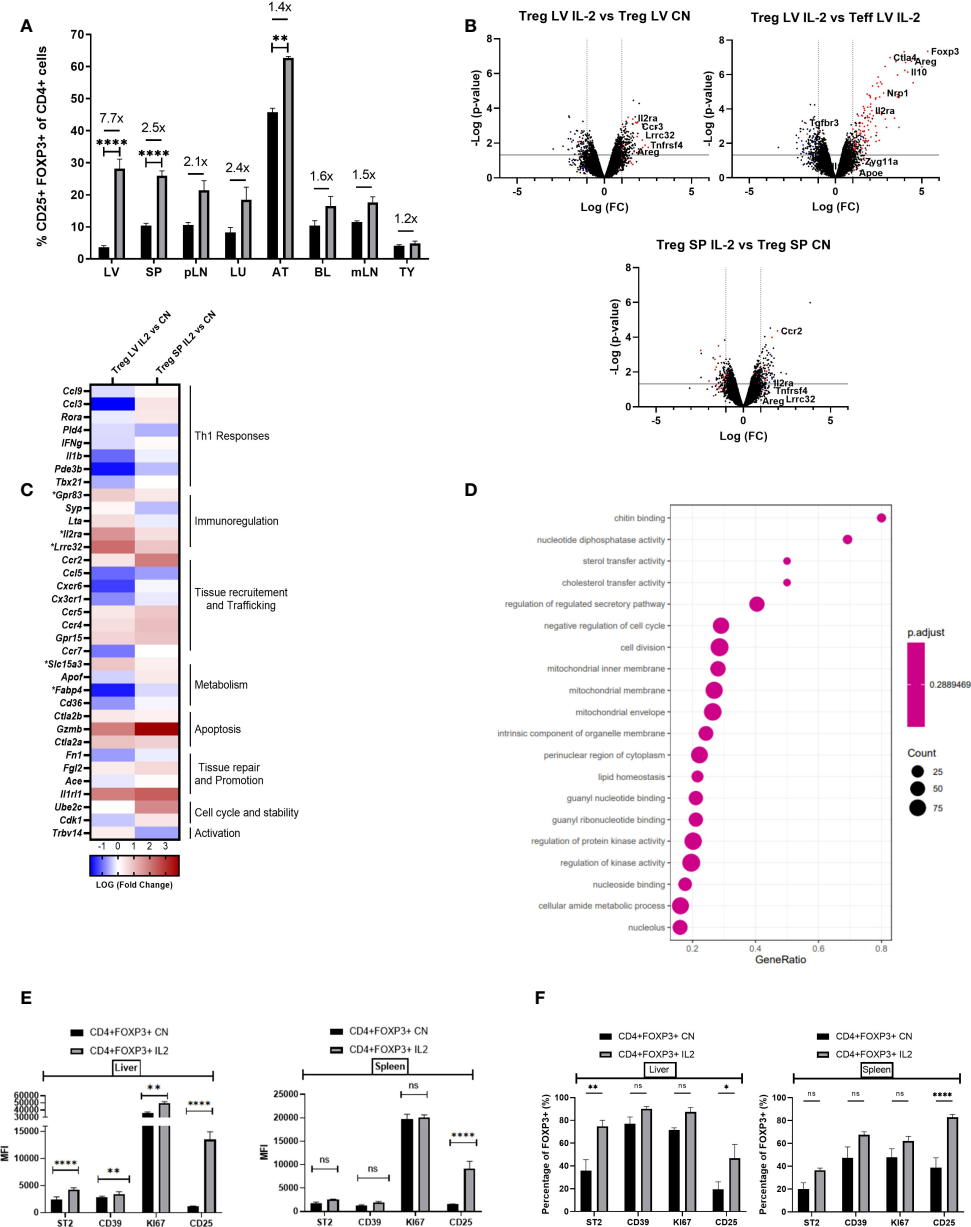
IL-2 administration increases Foxp3+ cells in the liver and enhances Treg signature transcriptional characteristics. **(A)** Quantification of the proportion of Foxp3+ CD4+CD25+ cells in the liver (LV) (n>8), spleen (SP) (n>8), peripheral lymph nodes (pLN), lung (LU), adipose tissue (AT), blood (BL), mesenteric lymph nodes (mLN) and thymus (TY) (n=4). **(B)** Volcano plot shows difference in the transcriptome profile of intra-hepatic Tregs and T-effectors when IL-2 is administered in Foxp3YFP mice (n=3) using normalised p-value versus fold change of all 20515 genes and splenic “Treg signature” is defined by up-regulated (red) and down-regulated (blue) genes above p-value of 0.05 and Fold change 2. **(C)** Heatmap exhibits differences in the expression of pre-defined differentially expressed genes relevant to Treg function, tissue residency, tissue repair and metabolism between intra-hepatic and splenic Treg population following IL-2c administration, and the significance is determined above p-value of 0.05 and Fold-change 2 and represented by a *. **(D)** Gene set enrichment analysis of IL-2c treated intra-hepatic Tregs compared with the intra-hepatic Tregs in homeostasis. Dot plot shows the top 20 up-regulated GO pathways of biological processes, molecular functions and cellular components with an FDR adjusted q-value of 0.020506. Dot size represents the number of genes enriched in the pathway, and gene ratio represents the ratio of the count of core enrichment genes to count of pathway genes. **(E, F)** Flow cytometric analysis of the effect of IL-2 administration on the previously defined differentially expressed transcripts of CD4+Foxp3+ cells in liver and spleen tissue, expressed as MFI and percentage. Significance was determined by ordinary one-way ANOVA with Sidak’s multiple comparison test for **(A)** and two-way ANOVA with Tukey multiple comparison test for **(E, F)** and the values are shown as the mean ± SEM. For B-D, p<0.05 and Fold Change >2. MFI, geometric mean fluorescence intensity. Ns, non-significant *P < 0.05, **P < 0.01 and ****P < 0.0001.

Our next step was to determine the effects of IL-2c, an established therapeutic strategy to expand the Treg compartment, on the numbers and phenotypic characteristics of intra-hepatic Tregs. IL-2c administration increased the proportion of Tregs in all tissue compartments analysed, with the largest increased being observed in the liver (7.7-fold increase, from 3.65%to 28.14%; **Figure 3A**). Microarray gene expression analyses of intra-hepatic Tregs revealed that, in addition to promoting Treg expansion, IL-2c administration resulted in increased expression of various Treg-specific genes, such as *Il2ra* (CD25), *Lrrc32*, *Gpr83* and *Areg* (**Figures 3B, C**). GSEA revealed that IL-2c treatment resulted in an enrichment in pathways involved in the regulation of T cell activation, proliferation, and migration, along with pathways associated with mitochondrial integrity, biogenesis, and lipid homeostasis (**Figure 3D**). These transcriptional changes induced by IL-2c in intra-hepatic Tregs were not observed in the splenic Tregs isolated from the same animals. Flow cytometry experiments showed that, upon administration of IL-2c, intra-hepatic Tregs increased the expression of ST2, CD39 (ENTPD1) and KI67 as compared to splenic Tregs, which is in keeping with the transcriptional results outlined above. In contrast, CD25 expression increased both in intra-hepatic and in splenic Tregs (**Figures 3E**, **F**), although CD25 MFI was noted to be higher in the intra-hepatic Treg compartment. Taken together, these results are suggestive exogenous IL-2c administration led to increases in intra-hepatic Treg pool and reverted low expression of key immunoregulatory molecules of these cells.

### The expansion of intra-hepatic Tregs in response to IL-2c administration is associated with reduced inflammatory liver damage

**Figure 4:**
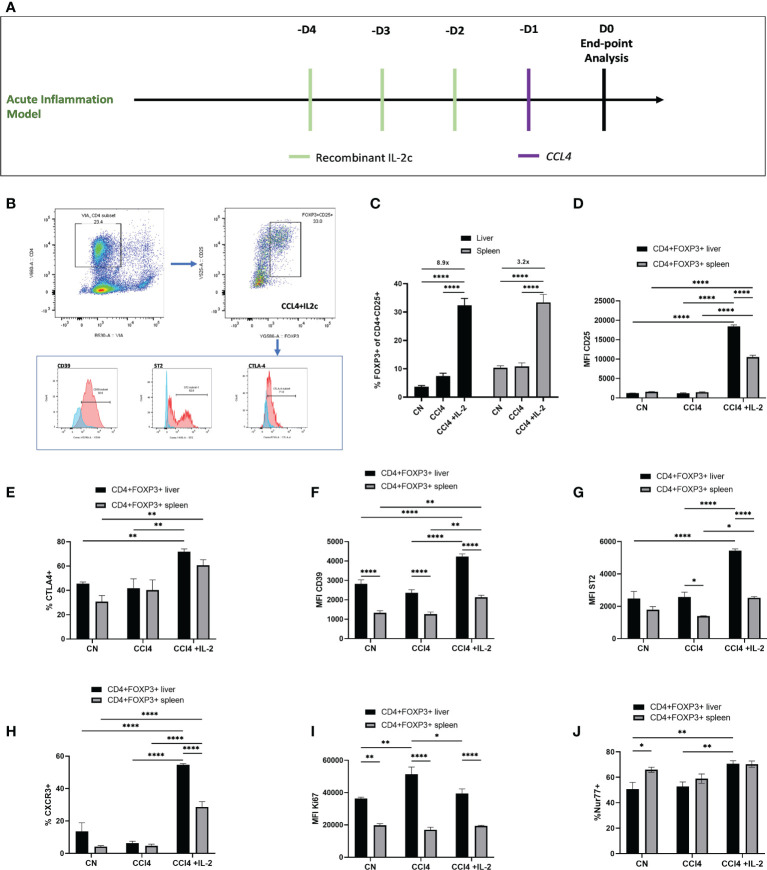
IL-2c expanded intra-hepatic Tregs exhibit enhanced expression of immunoregulatory transcripts in the inflammation rich microenvironment. **(A)** Schematic representation of the experimental design to generate CCL₄ induced acute liver inflammation; IL-2c administered for 3 consecutive days, following by a single dose of CCL₄ injection and animals were sacrificed 24h later for the tissue analysis. **(B)** Gating strategy employed to identify CD4+CD25+Foxp3+ cells and their expression profile, including the staining controls. **(C)** Percentage of CD4+CD25+Foxp3+ cells were quantified in liver and spleen in homeostasis, following IL-2c administration, CCL₄ induced inflammation and IL-2c administration prior to CCL₄ induced inflammation (n=9) along with **(D)** the changes in the MFI of CD25. Immunoregulatory profile and stability of these CD4+Foxp3+ cells in liver and spleen were characterised using flow cytometry by quantifying percentage of **(E)** CTLA4 expression and MFI of cell surface markers **(F)** CD39 and **(G)** ST2. **(H)** Percentage of Chemokine receptor CXCR3 positive cells regulating the homing of cells and **(I)** MFI of proliferating cells expressed by KI67+. **(J)** Additionally, percentage expression of NUR77 was compared between liver resident and splenic CD4+Foxp3+ cells. All data were quantified using flow cytometry (n=4). The values are shown as the mean ± SEM and one-way ANOVA with Tukey’s multiple comparison test has been performed to show statistical significance, Ns= non-significant *P < 0.05, **P < 0.01 and ****P < 0.0001.

Next, we explored the impact of acute liver inflammation induced by CCL₄, with or without IL-2c administration, on the phenotype of intra-hepatic Tregs (**Figures 4A, B**). The number of intra-hepatic Tregs increased 2-fold (from 3.65 ± 0.45% to 7.45 ± 0.99%) following injection of a single dose of CCl₄, with a further increase up to 8.9-fold when IL-2c was administered prior to CCL₄ (**Figure 4C**). CCL₄ did not influence the number of CXCR3+ Tregs or the expression levels of CD25, CD39, ST2 and CTLA4 (**Figures 4D–H**), but it increased intra-hepatic Treg proliferation (**Figure 4I**). In contrast, treatment with IL-2c prior to CCL₄ administration did not modify KI67 levels but it resulted in increased intra-hepatic Treg expression of CD25, CTLA4, CD39 and ST2, as well as in a marked expansion of intra-hepatic CXCR3+ Tregs (**Figures 4D–I**). We also observed that combined IL-2c plus CCL₄ treatment resulted in an increase in the intra-hepatic proportion of NUR-77-positive Tregs (**Figure 4J**), a marker of immediate TCR stimulation (28).

**Figure 5:**
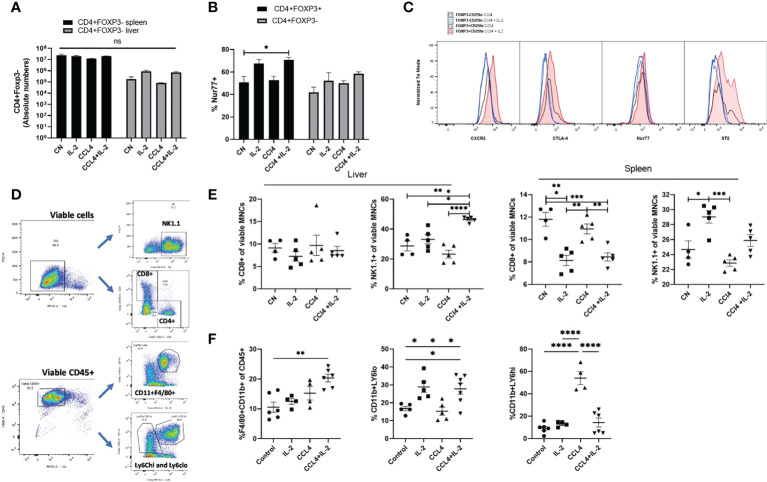
Characterisation of intra-hepatic cell subsets impacted by IL-2 administration and CCL₄ induced inflammation. **(A)** Absolute numbers of CD4+Foxp3- cells were quantified in liver and spleen in homeostasis, following IL-2c administration, CCL₄ induced inflammation and IL-2c administration prior to CCL₄ induced inflammation (n=5). **(B)** Percentage expression of NUR77 was compared between CD4+CD25+FOXP3+ Tregs and CD4+CD25+Foxp3- Effector T cells (n=4). **(C)** Additionally, these cell subsets were compared for changes in activation characterised by CTLA-4 and NUR77 expression and liver-specific regulation and homing seen by differences in ST2 and CXCR3 expression following CCL₄ alone or with IL-2 treatment, the plot is representative of 4 individual experiments **(D)** Gating strategy employed to characterise cell subsets. **(E)** Effect of CCL₄ induced liver injury with or without exogenous IL-2c on the percentage of CD8+ Effector T and NK cells in liver and spleen were summarised (n=4). **(F)** Changes in the hepatic non-parenchymal cell subsets were quantified by looking at the percentage abundance of Kupffer cells, Ly6C^hi^ pro-inflammatory and Ly6C^lo^ restorative macrophages following CCL₄ induced inflammation and exogenous IL-2c availability. The values are shown as the mean ± SEM and one-way ANOVA with Tukey’s multiple comparison test has been performed to show statistical significance. Ns, non-significant *P < 0.05, **P < 0.01, ***P < 0.001 and ****P < 0.0001.

In contrast to the effects of observed on intra-hepatic Tregs, neither IL2c nor CCL₄ increased the absolute numbers of intra-splenic and intra-hepatic CD4+Foxp3- Teffs, and in line with our transcriptome data, NUR-77, CTLA-4, CXCR3 and ST2 expression were not modified in CD4+Foxp3- Teffs following IL-2c administration (**Figures 5A–C**). Characterization of cell subsets (**Figure 5D**) showed that, neither IL-2c nor CCL₄ increased the number of intra-hepatic CD8+ cells, however a decrease in the splenic CD8+ percentage was observed with IL-2c administration, while an increase in NK and NKT cells was seen with IL-2c injection alone in spleen. Additionally, NK and NKT cells were increased by IL-2c administration in the inflamed liver compartment. (**Figure 5E**). Additionally, a shift to the pro-repair CD11b+Ly6C^lo^ M2 macrophage phenotype was observed in liver injury with the expansion of intra-hepatic Tregs. CCL₄ induced liver injury increased the percentage of pro-inflammatory CD11b+Ly6C^hi^ M1 macrophages, and this increase was reversed with IL-2c administration. Moreover, a decrease in the anti-inflammatory pro-restorative M2 macrophages was seen in the CCL₄ induced inflammation and this population was restored with IL-2c injections. Furthermore, we observed an increase in the percentage of CD11b+F4/80+ Kupffer cells in mice treated with IL-2c in the presence of CCL₄ induced inflammation. (**Figure 5F**).

**Figure 6:**
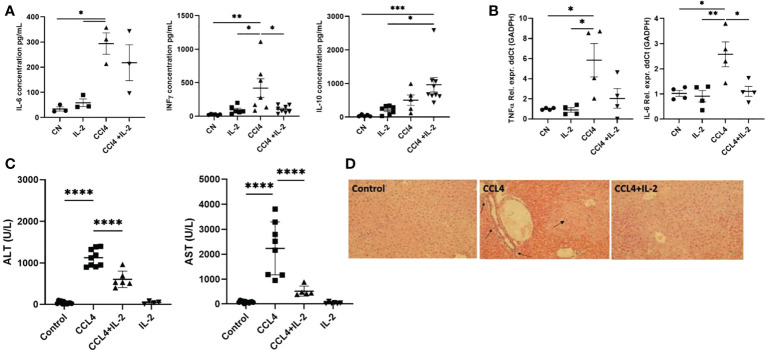
Impact of IL-2 administration on the modulation of inflammatory state of liver. Inflammation of liver was quantified by performing qPCR analysis on the RNA isolated from liver biopsies, assessing the distinctive cytokine profile and liver infiltrate in liver inflammation. **(A)** The quantification of pro and anti-inflammatory cytokines secreted in the serum (n=3) and **(B)** RNA extracted from the inflamed liver (n=4). **(C)** Levels of serum ALT and AST were measured at the study endpoint at 24h post-injection (n=5). **(D)** H&E staining of liver sections examined *via* light microscopy at 20x magnification. Black arrows indicate regions of excessive immune infiltration, and greater intensity of pink reveal regions of hepatocyte necrosis. The values are shown as the mean ± SEM and one-way ANOVA with Tukey’s multiple comparison test has been performed to show statistical significance *P < 0.05, **P < 0.01, ***P < 0.001 and ****P < 0.0001.

The changes in immune cell subsets induced by IL2c administration were associated with reduced expression of the pro-inflammatory cytokines *Il-6*, *Ifng*, and *Tnfa*, both in serum and in liver tissue, (**Figures 6A, B**), as well as with decreased AST/ALT serum levels (**Figure 6C**). These results were consistent with the histology analyses, which revealed that, in mice treated with CCL₄, IL-2c administration reduced both intra-hepatic leukocyte infiltration and hepatocyte necrosis (**Figure 6D**).

## Discussion

The cytokine IL-2, which is secreted by activated CD4 and CD8 T cells, is known to influence the differentiation, homeostasis, and effector properties of multiple immune cell subsets, but in particular of Tregs, whose function and survival are critically dependent on IL-2 availability (29). We previously described that in humans receiving calcineurin inhibitors, low IL-2 levels are responsible for the reduced number of circulating Tregs, which exhibit both increased proliferation and increased apoptosis (30). Likewise, lack of IL-2 has also been proposed as an explanation for the impaired function and pro-apoptotic tendency of Tregs isolated from human livers with end-stage inflammatory diseases (3). The results of our current study indicate that even under non-inflammatory quiescent conditions, intra-hepatic Tregs are reduced in number as compared to what is found in other organs and exhibit increased proliferation and features suggestive of lineage instability. Of note, while in the liver the proportion of Tregs among CD4+ T cells was the lowest of all tissues sampled, following administration of exogenous IL-2c intra-hepatic Tregs exhibited the greatest relative increase. This was associated with overexpression of molecules traditionally associated with Treg function and lineage stability. Taken together, these findings support the hypothesis that IL-2 deprivation is a major driver of the distinct features exhibited by intra-hepatic Tregs. The insignificant increase observed in the Treg numbers under inflammatory conditions could be associated with increased IL-2 production by the activated T cell infiltrate, demonstrating importance of IL-2 accessibility in controlling the frequency and survival of Tregs (31)

The liver microenvironment is known to be biased towards immune tolerance. Multiple mechanisms contribute to this state , including clonal deletion, clonal anergy and T cell exhaustion. The liver is known to trap activated T cells (e.g., autoreactive T cells), promoting their apoptotic clearance (32–34). Both parenchymal (e.g., hepatocytes, endothelial cells) and non-parenchymal cells contribute to this phenomenon (35–38). The extent to which different cell types and tolerogenic mechanisms are non-redundant and operate according to a universal pre-defined hierarchy, as opposed to being model dependant, remain unanswered questions. The data described in our report suggest that, in steady-state conditions, Tregs are unlikely to play a dominant role in maintaining intra-hepatic immune tolerance, given their low number and molecular programme. Likewise, the results of the DTR-Foxp3 CCL₄-induced liver damage model indicate that the small size of the intra-hepatic Treg pool exerts limited immunoregulatory effects in the setting of acute liver inflammation.

On the other hand, the increased expression levels of the IL-33 receptor (ST2 or IL1rl1) in intra-hepatic Tregs, both in homeostasis and following acute liver injury, suggests that Treg might be involved in liver regeneration and tissue repair, as previously described in muscle, lung, and intestine (12, 39, 40). IL-33 is released by activated endothelial cells in response to tissue damage. This results in the stimulation of ST2-positive Tregs, which then secrete amphiregulin, responsible for promoting tissue regeneration at least in part through the polarisation of monocytes towards an M2-like phenotype (41).

The expansion of the intra-hepatic Treg compartment in response to IL-2c significantly ameliorated liver damage following CCL₄ administration. The expansion was associated with increased transcript levels of *Ctla4* and CD39, whose expression is typically correlated with Treg function and fitness (42, 43). An important question to address is whether this expansion results from increased trafficking of circulating Tregs, or, alternatively, is secondary to the heightened proliferation and longevity of pre-existing tissue-resident Tregs. The fact that the administration of IL-2c to CCL₄-treated mice markedly increase the number of intra-hepatic CXCR3+ Tregs without modifying their KI67 levels, suggests that trafficking is the most likely mechanism. We hypothesize that this process is TCR-dependent, given the changes observed in NUR-77 levels, a marker of recent TCR stimulation (28). These data are in keeping with published literature demonstrating that the accumulation of Tregs in tissues is mostly dependent on their antigen specificity (44–46).

Our results are consistent with a recent report in a murine model of autoimmune hepatitis in which IL-2c ameliorated chronic inflammatory liver damage (47). Beyond animal models, low-dose recombinant IL-2 can effectively increase the number of circulating Tregs in humans and exert anti-inflammatory effects, as shown in multiple clinical trials in autoimmunity and in GVHD. Our group and others previously described the use of short courses of low-dose recombinant IL-2 to increase circulating Tregs in patients with autoimmune hepatitis (5) (48). In contrast to the results observed in autoimmunity, an additional study from our group in human liver transplantation revealed that low-dose IL-2 increased the immunogenicity of the liver allograft, facilitating rather than preventing allograft rejection (49). These somehow unexpected clinical results observed in liver transplant recipients are reminiscent of what has been described in murine models of intra-hepatic T cell priming, in which IL-2 reverts the inactivation of CD8+ T cells that takes place when they recognize cognate antigens expressed by hepatocytes (50). Recent data indicate that this process is regulated by NK and ILC1 cells, which constitute a significant proportion of intra-hepatic immune cells and compete with T cells for IL-2 (51). Altogether, these data indicate that IL-2 administration constitutes a double-edged sword in what regards controlling intra-hepatic inflammation.

Our study has a number of limitations that need to be taken into consideration to avoid over-interpretations. First, our experimental system does not allow to track antigen-specific Tregs or effector T cells. Second, the preferential binding of the of IL-2c composed of recombinant IL-2 and clone JES6-1A12 to the IL-2RA/B chains rather than the IL-2RBG is different than what is observed with recombinant IL-2 in humans. Third,we intentionally chose to investigate the role of Tregs in a model of acute inflammatory damage. While it would have been desirable to replicate similar experiments in a model of chronic liver damage, such a model would have been confounded by the marked systemic inflammatory effects mediated by auto-reactive T cells that results from chronic Treg depletion (52, 53). Previous work reported that the transient DT-induced Treg depletion in the late phase of chronic CCL₄ treatment resulted in aggravated liver damage and fibrosis (54), suggestive that with chronic damage, the situation could be different to our observations in acute damage. Additionally, our finding of a reduced proportion of Tregs in intra-hepatic CD4+ T cells would need to be confirmed in other murine strains and across a range of different ages. With these caveats in mind, we believe our report provides relevant novel insight into the complex immune network of liver microenvironment and the potential of intra-hepatic Tregs in modulating liver inflammation. These results will need to be taken into consideration when interpreting studies investigating strategies to boost or ameliorate liver inflammation both in animal models and in the clinic.

## Data availability statement

The datasets presented in this study can be found in online repositories. The names of the repository/repositories and accession number(s) can be found below: https://www.ncbi.nlm.nih.gov/, GSE80814.

## Ethics statement

The animal study was reviewed and approved by Animal Welfare and Ethical Review Body of King’s College London.

## Author contributions

MM-L supervised the project. AK, KS, PR-M, GO, TC, LD, KW, EG, SM, and EK performed and analyzed experiments. MM-L, NS and AS-F contributed to data analysis. AK, MM-L, AS-F and NS prepared the manuscript. All authors contributed to the article and approved the submitted version.

## Funding

This study was funded by the Medical Research Council (reference: MR/P007694/1).

## Conflict of interest

The authors declare that the research was conducted in the absence of any commercial or financial relationships that could be construed as a potential conflict of interest.

The reviewer JH declared a shared consortium with one of the authors, AS-F, to the handling editor.

## Publisher’s note

All claims expressed in this article are solely those of the authors and do not necessarily represent those of their affiliated organizations, or those of the publisher, the editors and the reviewers. Any product that may be evaluated in this article, or claim that may be made by its manufacturer, is not guaranteed or endorsed by the publisher.
