# Supplementary material for: IL-2 availability regulates the tissue specific phenotype of murine intra-hepatic Tregs
Source: Front Immunol. 2022 Oct 31;13:1040031. doi: 10.3389/fimmu.2022.1040031 (PMC9661520; doi:10.3389/fimmu.2022.1040031)
Supplement: Supplementary file 1 [file Table_1.docx]

**Table S1. Flow cytometry antibody list**

1. **Intrahepatic and intrasplenic immunophenotyping**

| Antibody Specificity | Clone | Fluorochrome | Manufacturer |
| --- | --- | --- | --- |
| LIVE/DEAD® Fixable Green Dead Cell Stain |  | FITC | Life Technologies |
| Anti-mouse CD3ε | 145-2C11 | APC Cy7 | Biolegend |
| Anti-mouse CD4 | RM4-5 | BV650 | Biolegend |
| Anti-mouse FOXP3 | 150D | PE | Biolegend |
| Anti-mouse CD25 | PC61 | BV510 | Biolegend |
| Anti-mouse CTLA4 | UC10-4B9 | PerCp Cy5.5 | Biolegend |
| Anti-mouse ST2 | DIH9 | BV421 | Biolegend |
| Anti-mouse CD39 | Duha59 | PE Cy7 | Biolegend |
| Anti-mouse Ki67 | 16A8 | BV605/PE Cy7 | Biolegend |
| Anti-mouse CD103 | 2E7 | PerCP Cy5.5 | Biolegend |
| Anti-mouse Nur77 | 12.14 | AlexaFluor 647 | BD Biosciences |
| Anti-mouse CD183 | CXCR3-173 | BV605 | Biolegend |
| Anti-mouse CD8a | 53-6.7 | PE Cy7 | Biolegend |
| Anti-mouse NK1.1 | PK136 | APC | Biolegend |
| Anti-mouse CD11b | M1/70 | BV421/APC Cy7 | Biolegend |
| Anti-mouse CD45 | 30-F11 | BV650/PE Cy7 | Biolegend |
| Anti-mouse F4/80 | BM8 | PerCP Cy5.5 | Biolegend |
| Anti-mouse I-A/I-E | M5/114.15.2 | PE | Biolegend |
| Anti-mouse Ly6C | HK1.4 | APC | Biolegend |
| Anti-mouse CX3CR1 | SA011F11 | BV711 | Biolegend |

**2. Cell Sorting**

| Antibody Specificity | Clone | Fluorochrome | Manufacturer |
| --- | --- | --- | --- |
| LIVE/DEAD® Fixable Violet Dead Cell Stain | - | V450 | Life Technologies |
| Anti-mouse CD3ε | 145-2C11 | APC-Cy7 | Biolegend |
| Anti-mouse CD4 | GK1.5 | PE-Cy7 | Biolegend |
| Anti-mouse CD25 | PC61 | APC | Biolegend |
| FOXP3 | - | FITC | YFP-cre |
