# Supplementary material for: IL-2 availability regulates the tissue specific phenotype of murine intra-hepatic Tregs
Source: Front Immunol. 2022 Oct 31;13:1040031. doi: 10.3389/fimmu.2022.1040031 (PMC9661520; doi:10.3389/fimmu.2022.1040031)
Supplement: Supplementary file 2 [file DataSheet_1.docx]

**Fig S1. Gating strategies employed to classify CD4+CD25+FOXP3+ cells and quantification of these cells in liver and spleen. (A)** Gating strategy employed to identify CD4+CD25+FOXP3+ cells and **(B)** Treg frequencies in various liver and spleen samples from various independent experiments.


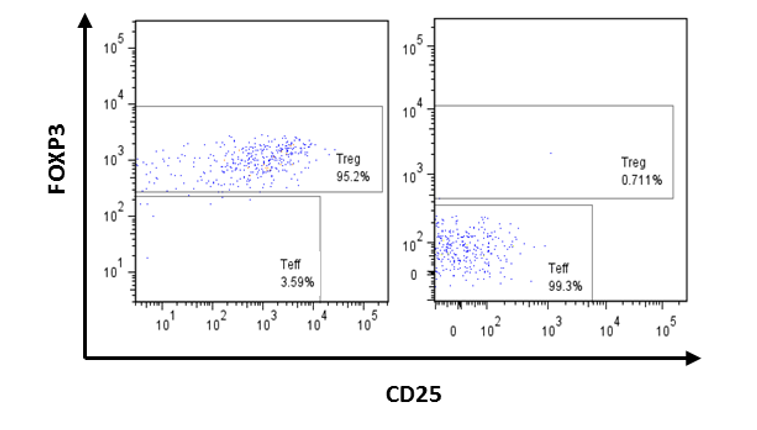


**Fig S2. Treg/Teff isolation purity following T cell sorting based on YFP reporter.**

**Fig S3. Absolute number of Tregs per million of events acquired.** Absolute numbers of CD25+FOXP3+ cells were quantified in liver and spleen in homeostasis, following IL-2c administration. CCL4 induced inflammation and IL-2c administration prior to CCL4 induced inflammation (n=5). The values are shown as the mean ± SEM and one-way ANOVA with Tukey’s multiple comparison test has been performed to show statistical significance. * p < 0.05, ** p <0.01, *** p < 0.001 and **** p < 0.0001
